# Preliminary Characterisation of Tumor Necrosis Factor Alpha and Interleukin-10 Responses to *Chlamydia pecorum* Infection in the Koala (*Phascolarctos cinereus*)

**DOI:** 10.1371/journal.pone.0059958

**Published:** 2013-03-19

**Authors:** Marina Mathew, Kenneth W. Beagley, Peter Timms, Adam Polkinghorne

**Affiliations:** Institute of Health & Biomedical Innovation, Queensland University of Technology, Kelvin Grove, Brisbane, Australia; Auburn University, United States of America

## Abstract

Debilitating infectious diseases caused by *Chlamydia* are major contributors to the decline of Australia's iconic native marsupial species, the koala (*Phascolarctos cinereus*). An understanding of koala chlamydial disease pathogenesis and the development of effective strategies to control infections continue to be hindered by an almost complete lack of species-specific immunological reagents. The cell-mediated immune response has been shown to play an influential role in the response to chlamydial infection in other hosts. The objective of this study, hence, was to provide preliminary data on the role of two key cytokines, pro-inflammatory tumour necrosis factor alpha (TNFα) and anti-inflammatory interleukin 10 (IL10), in the koala *Chlamydia pecorum* response. Utilising sequence homology between the cytokine sequences obtained from several recently sequenced marsupial genomes, this report describes the first mRNA sequences of any koala cytokine and the development of koala specific TNFα and IL10 real-time PCR assays to measure the expression of these genes from koala samples. In preliminary studies comparing wild koalas with overt chlamydial disease, previous evidence of *C. pecorum* infection or no signs of *C. pecorum* infection, we revealed strong but variable expression of TNFα and IL10 in wild koalas with current signs of chlamydiosis. The description of these assays and the preliminary data on the cell-mediated immune response of koalas to chlamydial infection paves the way for future studies characterising the koala immune response to a range of its pathogens while providing reagents to assist with measuring the efficacy of ongoing attempts to develop a koala chlamydial vaccine.

## Introduction

The koala (*Phascolarctos cinereus*) is an Australian arboreal marsupial and the last surviving member of the family *Phascolarctidae*. Despite enjoying a unique position in Australian culture, the koala faces the threat of localised extinction in many regions along the Australian eastern seaboard [1], [2], [3]. The recognition of this decline has recently lead to its inclusion in the threatened species list by the Australian Government Department of Sustainability, Environment, Water, Population and Communities [4]. Several threats are contributing to this decline, including habitat destruction [1], natural disasters [5], dog attacks [6] motor vehicle accidents [7] and disease. Of the many threatening processes affecting the koala, infectious diseases appear to have the greatest impact with a recent study showing that if diseases in this host could be reduced by up to 50%, then declining koala populations had the potential to be stabilised [8].

*Chlamydia pecorum* is the main aetiological agent of infectious disease in the koala with studies reporting around 60–98% prevalence of chlamydial infection in certain free range koala populations [9], [10], [11], [12]. *C. pecorum* infection in koalas has been associated with cystitis, proliferative conjunctivitis and chronic, fibrotic disease of the urogenital tract leading to infertility and death [13], [14], [15], [9], [16], [12]. In non-koala hosts, the role of cell mediated immunity (CMI) as the major immune response generated by the host against chlamydial infection is acknowledged [17], [18], [19]. Inflammation, driven by the effects of inflammatory cytokines, has also been linked to the manifestation of disease [20].

A major hurdle in our understanding of the role of the koala immunological response in relation to these diseases is the paucity of immunological reagents for this host. In their absence, current assays employed to characterise the marsupial immune response rely on reagents from placental mammals, which exhibit only weak cross-reactivity in marsupial systems [21]. The ideal targets for measuring local and systemic immune response to intracellular pathogens are cytokines; soluble immune regulators produced by cells of both the innate and adaptive immune systems. In this study, we aimed to develop koala-specific quantitative real time PCR (qrtPCR) assays and perform preliminary studies on the expression of two key cytokines, pro-inflammatory cytokine, TNFα, as well as a key anti inflammatory cytokine, IL10. TNFα is a trimeric pro-inflammatory polypeptide synthesised principally by macrophages in response to bacterial toxins, inflammatory products and other invasive stimuli [22]. IL10 is a dimeric polypeptide secreted by cells of the innate and adaptive immune system, the main function of which is to limit and ultimately terminate inflammatory responses [23], [24]. The partial sequence of a house-keeping gene, glyceraldehyde 3-phosphate dehydrogenase (GAPDH), was also determined for use as an internal control. qrtPCR assays were then developed to measure these two key cytokines within koala peripheral blood mononuclear cells (PBMCs) revealing strong expression in koalas with current signs of chlamydial disease.

## Materials and Methods

### Ethics statement

The collection of blood samples from koalas admitted to Australia Zoo Wildlife Hospital, Beerwah was performed by qualified veterinarians. Blood was collected from all animals following anaesthesia for other routine veterinary procedures and treatment. The collection and subsequent analysis of these koala blood samples was approved by the Queensland University of Technology Animal Ethics Committee (Approval No. 0700000845).

### Bioinformatics analysis of koala cytokine sequences

Multiple sequence alignments were carried out using the Geneious Alignment program in the Geneious Pro 5.6.5 software and GeneDoc version 2.7.000 software. Phylogenetic trees were constructed using Jukes Cantor, UPGMA tree build method in Geneious Pro 5.6.5 software. TNFα and IL10 sequences were obtained from GenBank (GenBank ID): brushtail possum IL10 (AF026277), North American opossum IL10 (XM_003340167), mouse IL10 (M37897), sheep IL10 (Z29362), deer IL10 (U11767), horse IL10 (EU438771), cat IL10 (AF060520), monkey IL10 (DQ890063), human IL10 (AY029171), dog IL10 (NM_001009209), chicken IL10 (AY647438), Pigeon IL10 (AB618540), brushtail possum TNFα (AF016102), North American opossum TNFα (AJ286832), tammar wallaby TNFα (AF055915), red kangaroo TNFα (AJ286833), mouse TNFα (EU682384), sheep TNFα (NM_001024860), deer TNFα (U14683), horse TNFα (NM_001081819), cat TNFα (NM_001009835), monkey TNFα (DQ902477), human TNFα (AB451492), dog TNFα (NM_001003244) and chicken TNFα (AY765397).

### Sample collection

PBMCs were harvested from koala blood samples for use in this study. 5–6 ml of blood was collected in 6 ml EDTA blood tubes from koalas brought into the hospital and stored at 4°C until further processing on the same day. Swabs were collected from the conjunctiva of the left eye, right eye, urogenital sinus (females) and urethra (males) using aluminium shafted cotton tipped swabs (Copan, Interpath Services, Melbourne).

### Koala lymphocyte stimulation assay

PBMCs from the blood samples were separated using Ficoll Paque gradient centrifugation at 400 g for 20 mins (GE Healthcare, Rydalmere, Australia). After 2–3 washings with PBS, the cells were suspended in 1 ml of RPMI 1640 T cell media, supplemented with 5% foetal calf Serum, antibiotics and β-mercaptoethanol (0.001 M). The cells were then diluted to a concentration of 2×10^6^ cells/ml in the media and mixed with an equal volume of T cell mitogens, PMA (1 µg/ml) and Ionomycin (50 ng/ml) to stimulate koala PBMCs. To optimise the target gene expression, PBMCs were stimulated for different durations of time (24, 48 and 72 hours). The cell suspension and mitogen were added at a 1:1 concentration for a final volume of 200 µl/well to standard 96-well microtitre plates (Nunc) and incubated at 37°C in 5% CO_2_. At the end of the incubation period, cells were harvested from the cell suspension by centrifugation at 400 g for 10 mins and suspended in 1 ml of Trizol reagent (Invitrogen, Victoria, Australia) and left at room temperature for 5 mins to homogenise. Samples were then stored at −80°C for downstream applications.

### RNA extraction and reverse transcription

RNA extraction was performed from the PBMCs suspended in Trizol reagent using the RNeasy Mini Kit (Qiagen, Victoria, Australia) according to the manufacturer's instructions. The concentration and purity of RNA was determined using a Nanodrop ND-1000 Spectrophotometer. RNA degradation was checked by running the sample on a 2% agarose gel with the presence of two distinct bands depicting 18S and 28S rRNA indicating the quality of RNA isolation and absence of any degradation. In order to eliminate any possible DNA contamination, the RNA preparation was subjected to DNase digestion using Amplification Grade Deoxyribonuclease I (Sigma Aldrich, NSW, Australia) and immediately processed for cDNA synthesis. The reverse transcription reaction using random hexamer primers was performed using the Transcriptor First Strand cDNA Synthesis Kit (Roche, NSW, Australia) according to the manufacturer's instructions. The cDNA obtained was used as template for conventional PCR and qrtPCRs.

### Koala cytokine sequence cloning and sequencing

Primers for koala cytokine gene detection were designed based on alignment of homologous gene sequences from other species. A sequence alignment was carried out for the coding sequence of each gene from various species accessioned in Genbank, with *Trichosorus vulpecula* (Australian common brushtail possum) as the reference sequence for primer design. For IL10 primer design, brushtail possum, dog, cat, horse, pig, human, rat, rabbit and mouse IL10 sequences were used for alignment construction. Similarly, for TNFα primer design, brushtail possum, red kangaroo and wallaby TNFα sequences were used. The primers were designed along highly conserved regions to maximise chances of amplifying the corresponding gene in the koala (data not shown).

Upon successful amplification, PCR product of the expected size was cloned into a plasmid using Promega pGEM-T Easy Vector Systems I. In order to validate the sequences, a minimum of five clones were grown overnight at 37°C in LB medium (Luria-Bertani Medium) with ampicillin (100 µg/ml) with intermittent shaking. DNA was extracted using the Purelink Quick Plasmid Miniprep Kit (Invitrogen, Victoria, Australia). Success of cloning was determined by digesting the plasmid DNA using Restriction Endonuclease *Eco*R I (Roche, Applied Science, Germany). A single digest with EcoR1 releases inserts cloned into the pGEM-T Easy Vector. The digested product was then run on a 1.5% agarose gel along with the uncut plasmid. The presence of a band of the expected size indicated successful insertion of the target sequence into the vector. The products were then sequenced at Australian Genome Research Facility using the AB 3730*xl* platform.

### Design and optimisation of koala cytokine quantitative SYBR Green I RT-PCR assays

**Figure 1:**
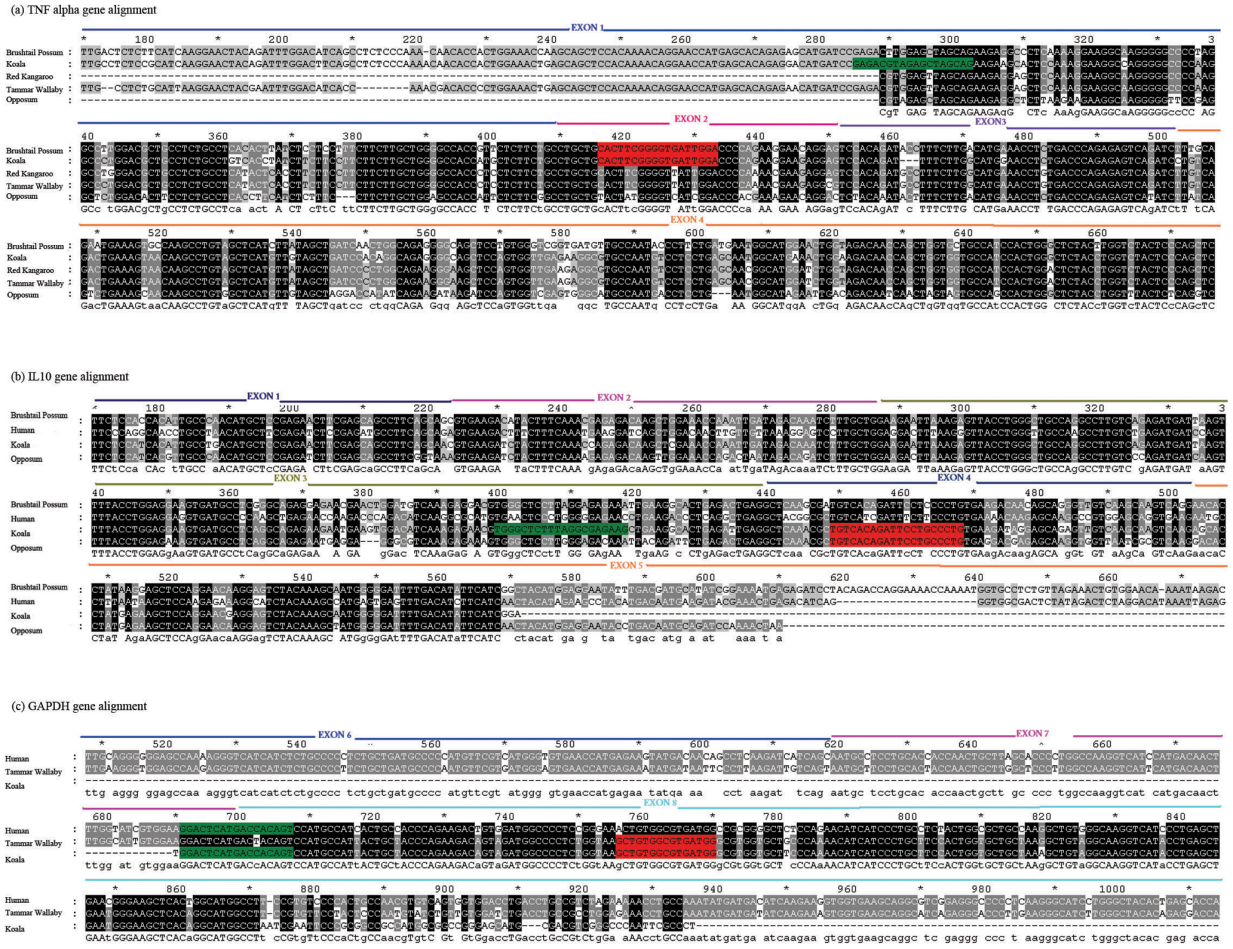
The exon distribution and primer position of koala TNFα (a), IL10 (b) and GAPDH (c) genes when in alignment with other marsupial and human sequences of the corresponding genes. The forward and reverse primer sequences used in the RT-PCR assays are highlighted in green and red, respectively. The PCR product has been designed such that it spans over an intron-exon junction.

Koala TNFα and IL10 cytokine mRNAs identified as part of this study were used to design primers for the qrtPCR assay using Primer Express® Software v3.0 by Applied Biosystems targeting 180 bp and 72 bp regions, respectively. The sequences for each primer used in qrtPCR assays are summarised in Table 1. Furthermore, primers which would amplify a PCR product spanning over two postulated exons based on comparison of these locations in tammar wallaby or human sequences were designed in order to avoid any co-amplification of contaminating genomic DNA in the reaction mixture (Figure 1).

**Table 1:** qrtPCR primers for TNFα, IL10 and GAPDH genes.

Gene	Forward Primer 5′–3′	Reverse Primer 5′–3′	Target
TNF α	GAGACGTAGAGCTAGCAG	TGCCAAGAAAATCTGTGGAC	180 bp
IL 10	TGGGCTCTTTAGGCGAGAAG	CAGGGCAGGAATCTGTGACA	72 bp
GAPDH	GGACTCATGACCACAGT	CCATCACGCCACAGC	70bp

The target cytokine genes were optimised into qrtPCR assays using SYBR Green I dye chemistry for gene quantification at the mRNA level. Assay efficiency was determined by constructing standard curves for the two cytokines using purified PCR product serial dilutions of known concentration at 10^2^, 10^4^, 10^6^, 10^8^ gene copy numbers. Conventional PCR with each gene-specific primer pair was used to prepare serially diluted qrtPCR standards (10^2^, 10^4^, 10^6^ and 10^8^). Approximately 100 µl of the amplified PCR product was run on a 1.5% agarose/TBE gel to confirm successful amplification. Corresponding bands were excised from the gel and solubilised using the High Pure PCR Product Purification Kit (Roche, Applied Science, Germany) as per the manufacturer's instructions. Purified PCR product was checked for quality and concentration using Nanodrop ND-1000 Spectrophotometer at absorbance of 260 and 280 nm wavelengths. The number of molecules of the product was calculated using Avagadro's formula.

All reactions were carried out on a Corbett Rotor Gene 6000 real time PCR machine at a final volume of 20 µl, with 1 unit of Fastart Taq Polymerase (Roche), 2 µl of 25 mM MgCl_2_ (Roche), 2 µl of 10 x buffer (Roche), 1 µl of 10 mm dNTPs (Roche), 3 µl of 1/10000 SYBR Green and 0.6 µl each of 5 mM forward and reverse primers. After an initial incubation of 95°C for 10 mins, 40 cycles of 10 s at 95°C, 25 s at 56°C and 15 s of 72°C were carried out for IL10 qrtPCR. For TNFα qrtPCR, after an initial incubation of 95°C for 10 mins, 40 cycles of 10 s at 95°C, 20 s at 54°C and 15 s of 72°C were carried out. All samples were tested in triplicate. Reactions were carried out at a final volume of 20 µl. Mastermix with no cDNA was used as the no template control whereas water was used as the negative control.

In order to facilitate comparison between animals and experiments and to normalise the variable amount of starting material used for each experiment, GAPDH, a house-keeping gene, was chosen as reference gene for the qrtPCR assay. The koala GAPDH gene was isolated using consensus primers designed from available GAPDH gene sequences. The real time GAPDH primers targeted a 70 bp region for which standards of known concentrations of 10^2^, 10^4^, 10^6^ and 10^8^ copy numbers were prepared as previously described. The reactions were carried out in 20 µl final volume. An initial incubation of 95°C for 10 mins was followed by 40 cycles of denaturation for 10 s at 95°C, 15 s annealing at 52°C and 20 s extension at 72°C for GAPDH qrtPCR assay. The target cytokine genes were normalised to GAPDH by the 2^−ΔΔCT^ method, where ΔΔCT  =  (Ct of target – Ct of GAPDH) at any time point – (Ct of target – Ct of GAPDH) at time zero hour [25].

### TNFα and IL10 mRNA production in koala PBMCs upon in vitro exposure to chlamydial antigens

In order to determine TNFα and IL10 production levels in koalas upon exposure to chlamydial antigens, blood samples were collected from 10 koalas brought into Australia Zoo Wildlife Hospital. PBMCs were processed from the blood samples and cultured in the presence of UV inactivated *C. pecorum*. A 500 µl stock of semi-purified *C. pecorum* G [26] was UV inactivated as described by Rey-Ladino et al. [27] and further diluted 1/20 dilution in RPMI 1640 T cell media. The plasma samples from these animals were stored at −80°C for use in western blot analysis. Cells were then cultured in the presence of UV inactivated *C. pecorum* G at 1:1 ratio for durations of 12, 24 and 48 hours. Unstimulated cells were collected at the start of the experiment to calculate baseline data for qrtPCR assays. At the end of the stimulation periods, RNA extraction and cDNA synthesis was performed and TNFα, IL10 and GAPDH mRNA expression was determined using our koala specific qrtPCR assays, designed as part of this study.

### *C. pecorum* species specific PCR screening and Western Blot analysis

To determine the current and previous *C. pecorum* infection status of koalas analysed as a part of this study, swab samples (eyes, urogenital tract) and blood samples were collected from 10 koalas that presented to the Australian Wildlife Hospital for veterinary care (Table 2).

**Table 2:** Clinical presentation and *C. pecorum* infection status for animals analysed as a part of this study.

Animal ID	Clinical Presentation	*C. pecorum* species -specific qrtPCR result	*C. pecorum* MOMP Western Blot
**Group I (n = 4)**			
#42983	Cystitis	Positive	Positive
#42096	Cystitis	Positive	Positive
#42082	Conjunctivitis	NEGATIVE	Positive
#42980	Cystitis	NEGATIVE	Positive
**Group II (n = 3)**			
#40520	Dog attack	NEGATIVE	Positive
#42097	Vehicle hit	NEGATIVE	Positive
#38940	Gut disease	NEGATIVE	Positive
**Group III (n = 3)**			
#42012	Vehicle hit	NEGATIVE	NEGATIVE
#42943	Vehicle hit	NEGATIVE	NEGATIVE
#53470	Healthy	NEGATIVE	NEGATIVE

The presence of *C. pecorum* DNA in koala swabs was determined using a previously described *C. pecorum* species-specific qrtPCR [28] targeting a 202 bp region of the 16S rRNA region using primers 16Sf (5′ – AGTCGAACGGAATAATAATGGCT –3′) and 16Sr (5′ – CCAACAAGCTGATATCCCAC –3′). Briefly, DNA was extracted from swab samples following vortexing and centrifugation and then used as a template in the qrtPCR. 25 µl qrtPCR reactions were performed in duplicates with 3 µl of extracted DNA template, 1 unit of Fastart Taq Polymerase (Roche), 2.5 µl of 25 mM MgCl_2_ (Roche), 2 µl of 10 x buffer (Roche), 0.6 µl of 10 mm dNTPs (Roche), 3 µl of 1/10000 SYBR Green and 0.6 µl each of 10 mM forward and reverse primers. Reactions were carried out in a Corbett Rotor Gene 6000 real time PCR machine with an initial denaturation at 95°C for 5 mins, followed by 94°C for 30 s, annealing at 57°C for 15 s and extension at 72°C for 25 s for 40 cycles. Standards with 10^8^, 10^6^, 10^4^ and 10^2^ of *C. pecorum* 16S rRNA genomic DNA copy numbers were used as calibrators for the assay. High resolution melt analysis was performed to confirm the *C. pecorum* specific PCR product with a melting temperature range between 82°C and 85°C [29].

Western blots were performed using recombinant *C. pecorum* His-tagged major outer membrane protein (MOMP) A, F and G antigens as described by Kollipara et al. [30] to detect the presence of *C. pecorum* MOMP-specific koala IgG antibodies in the koala plasma samples.

### Statistics

Graph-Pad Prism version 5 (Graph Pad Software, Lajolla, CA, USA) was used to perform statistical analyses. Unpaired *t*- test with the *P* value set at <0.05 was used to analyse significance of TNFα and IL10 mRNA expression relative to GAPDH among the different groups of koalas analysed.

## Results

### Koala cytokine mRNA sequence identification and analysis

This is the first study to report the sequences of any koala cytokine. As the starting point for this study, consensus primers were designed against partial sequences for the TNFα gene from brushtail possum, red kangaroo and wallaby. Similarly, for IL10 primer design, brushtail possum, dog, cat, horse, pig, human, rat, rabbit and mouse IL10 sequences were used. Following successful PCR amplification and sequencing of the cloned PCR products, 958 bp of koala TNFα (GenBank accession number – JX845580) and 479 bp of koala IL10 (GenBank accession number – JX845581) mRNA were identified (Figure 1A and 1B). BLAST analysis of available sequences and comparison of the TNFα sequence in the koala with sequences from other marsupials, mammals and avians, shows that, not surprisingly, the koala sequences are more similar to the red kangaroo (90.1%), tammar wallaby (88.7%), North American opossum (80.7%) and brushtail possum (84.4%) sequences than they are to the mammalian or avian TNFα sequences. A comparison of the koala IL10 sequence revealed a similar picture, with the highest homology observed with the marsupial species sequences available from, brushtail possum (91%) and North American opposum (83.9%), compared to the mammalian or avian species.

**Figure 2:**
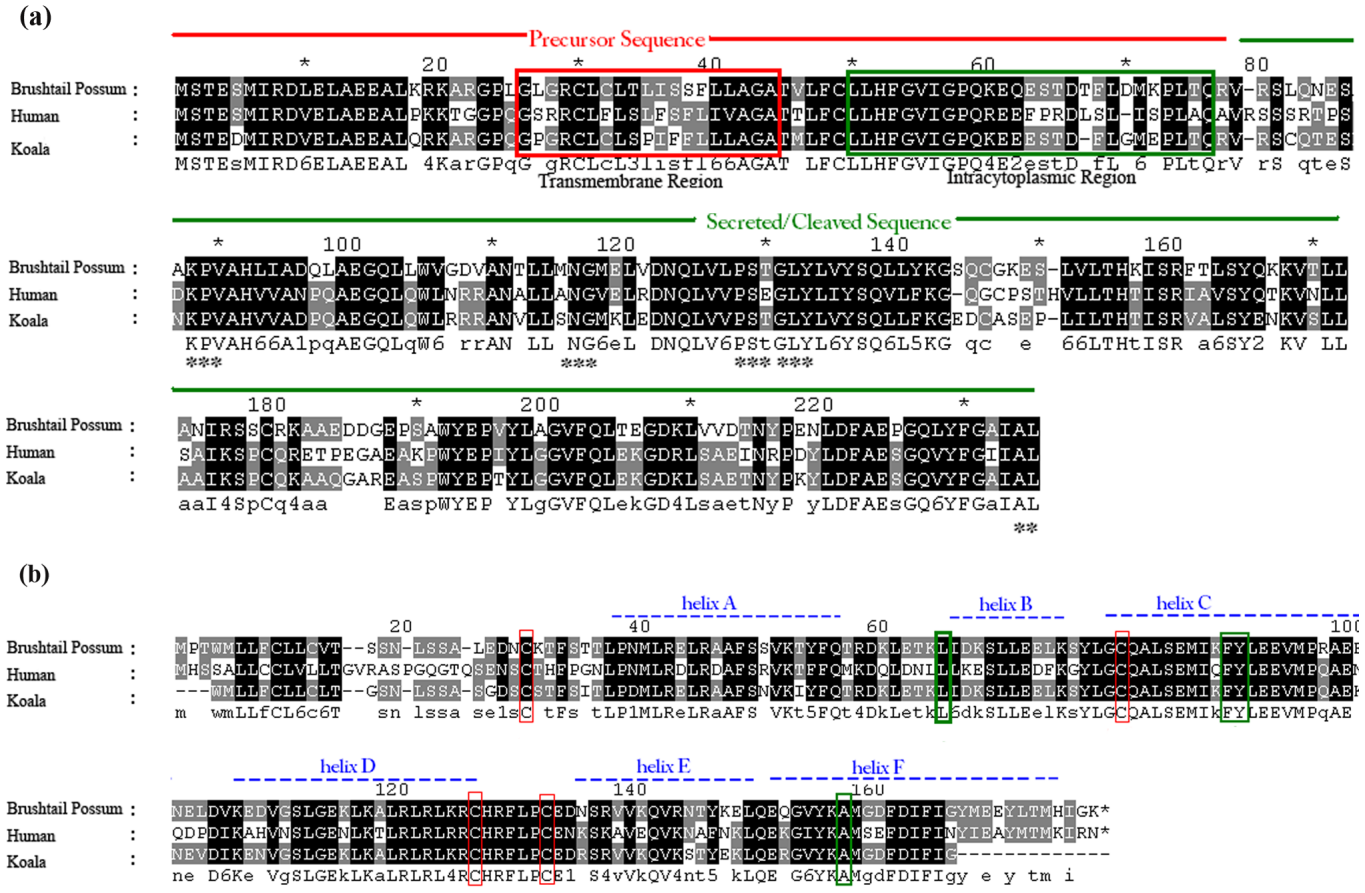
Amino acid sequence alignment of koala, brushtail possum and human TNFα sequences. (a) Koala TNFα sequence characterisation is done with reference to the human TNFα sequence. Presequence comprising of 76 amino acids and the secreted form of 156 amino acids is indicated by the horizontal red and green lines respectively. The hydrophobic transmembrane region and the intracytoplasmic region are marked out in red and green boxes respectively [31]. Asterisks indicate the receptor binding sites of the protein [32]. (b) Koala IL10 sequence characterisation is done with reference to the human IL10 sequence. Cysteine residues which form intramolecular disulphide bonds in human IL10 are marked in red boxes. Residues important for structural stabilisation are marked in green boxes. The six α-helices of the IL10 monomer are depicted by dotted blue lines [33].

The total length of TNFα mRNA in humans and tammar wallaby is 1669 bp (GenBank: NM_000594) and 1771 bp (GenBank: AY853666) respectively. The tammar wallaby TNFα gene consists of 4 exons and 3 introns. We cloned 958 bp of the koala TNFα nucleotide sequence which corresponds to the entire first, second and third exons of the sequence as well as approximately 65% of exon 4 (Figure 1A). This covers the entire 5′ untranslated region and the complete coding sequence for the protein [31]. An analysis of the koala TNFα amino acid sequence with reference to the human TNFα reveals several structural similarities (Figure 2A). The predicted transmembrane and intracytoplasmic regions show considerable homology [31] and the receptor binding sites are highly conserved as well [32].

The complete mRNA of brushtail possum IL10 is 1604 bp long (GenBank: AF026277) which has 91% similarity to the 479 bp of the cloned IL10 koala sequence. Of the five exon regions of the brushtail possum IL10 gene, the available koala sequence spans exons 1, 2, 3, 4 and 5 (Figure 1B). The koala IL10 sequence cloned as part of this study lies within the coding sequence for the protein when compared to the brushtail possum IL10 coding sequence. The brushtail possum IL10 coding sequence translates to a 175 amino acid protein, whereas the koala IL10 sequence translates to a 159 amino acid sequence. An evaluation of the koala IL10 amino acid sequence with reference to the human IL10 sequence reveals conservation of several key features. Four cysteine residues that form intramolecular disulphide bridges in human IL10 protein are conserved in the deduced koala IL10 sequence (Figure 2B). Similarly, residues that are important for IL10 structural stabilisation [33] are also conserved across the two species (Figure 2B).

**Figure 3:**
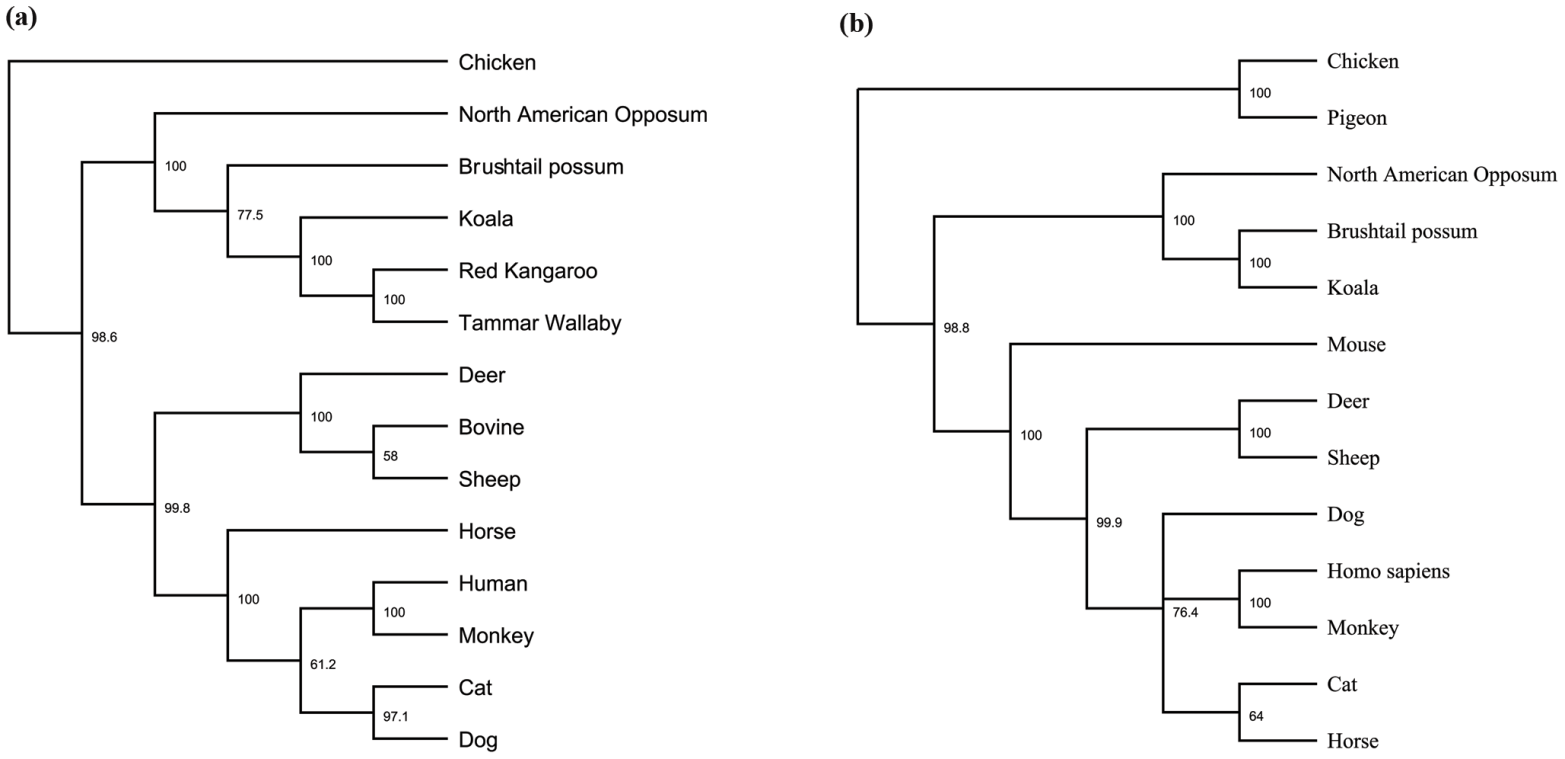
Phylogenetic tree of (a) TNFα and (b) IL10 nucleotide sequence constructed using Geneious Pro 5.6.5 using Jukes Cantor, UPGMA tree build method with 1000 bootstrap repeat value.

The relationship of the koala TNFα and IL10 sequences with other marsupial and mammalian TNFα and IL10 sequences is illustrated in a phylogenetic tree (Figures 3A and 3B). It shows that the marsupial, mammalian and avian TNFα and IL10 sequences have evolved along different lines, with the koala sequences forming a distinct clade with other marsupial sequences, away from placental mammals and birds.

### Development of koala cytokine qrtPCR assays

qrtPCR assays were developed using koala TNFα, IL10 and GAPDH sequences to measure their expression in koala peripheral blood samples. The primers for all three genes were designed such that the amplicons would span over at least one intron-exon junction, thereby eliminating any chances of amplifying contaminant DNA left in the sample. R^2^ values for all assays were found to be 0.99. The C_T_ value of the standards used was found to be similar in consecutive runs, thereby confirming the reproducibility of the assay. Moreover, agarose gel visualisation of representative replicates from RT-PCR reactions revealed appropriately-sized bands. Efficiency of the reaction was calculated as 10^(−1/slope)^. All three assays achieved greater than 90% efficiency and could detect <10^2^ gene copies. These values illustrate that the RT-PCR assay for the TNFα, IL10 and GAPDH gene are optimised and can be used for quantification of these genes in an unknown sample.

### TNFα and IL10 expression in koala PBMCs stimulated with UV- inactivated *C. pecorum*

To provide preliminary data on the koala TNFα and IL10 response to chlamydial infection, blood samples were collected from 10 koalas presenting to the Australia Zoo Wildlife Hospital for veterinary care. Based on the results and the presence of overt signs of koala chlamydiosis, the 10 koalas were divided into three groups: Group I (n = 4) – koalas with current clinical signs of chlamydiosis; Group II (n = 3) – *C. pecorum* PCR and/or western blot positive koalas without overt signs of chlamydiosis; and Group III (n = 3) – PCR and/or western blot negative koalas with no overt signs of chlamydiosis (Table 2).

Four koalas (Group I) included in this analysis presented with signs consistent with active chlamydial infection (i.e. conjunctivitis and/or cystitis) whereas the remaining six animals were brought into the hospital for conditions other than chlamydial infection (Table 2). Ocular and urogenital swabs were collected from all animals and screened for the presence of *C. pecorum* DNA using a species-specific 16S rRNA qrtPCR, as previously described (Wan *et al*., 2011).

Of the Group I animals, the urogenital tract swabs of two animals (#42983 and #42096) presenting with cystitis were found to be qrtPCR positive (31 and 588 16S rRNA copies/µL of extracted DNA, respectively), indicating mild to moderate shedding of *C. pecorum* from these anatomical sites (Wan et al., 2011). All animals in groups II and III were qrtPCR negative for *C. pecorum* DNA as was the remaining animals from Group I (#42082 and #42980). To evaluate previous exposure to a *C. pecorum* infection, the presence of koala-*C. pecorum* specific IgG antibodies was determined for all animals by Western blot using a panel of recombinant *C. pecorum* MOMPs. All four Group I animals were found to be positive for *C. pecorum* MOMP-specific IgG (data not shown). Of the remaining animals, three animals without overt signs of chlamydiosis were also found to be Western blot positive (Group II) while the rest were Western blot negative (Group III).

**Figure 4:**
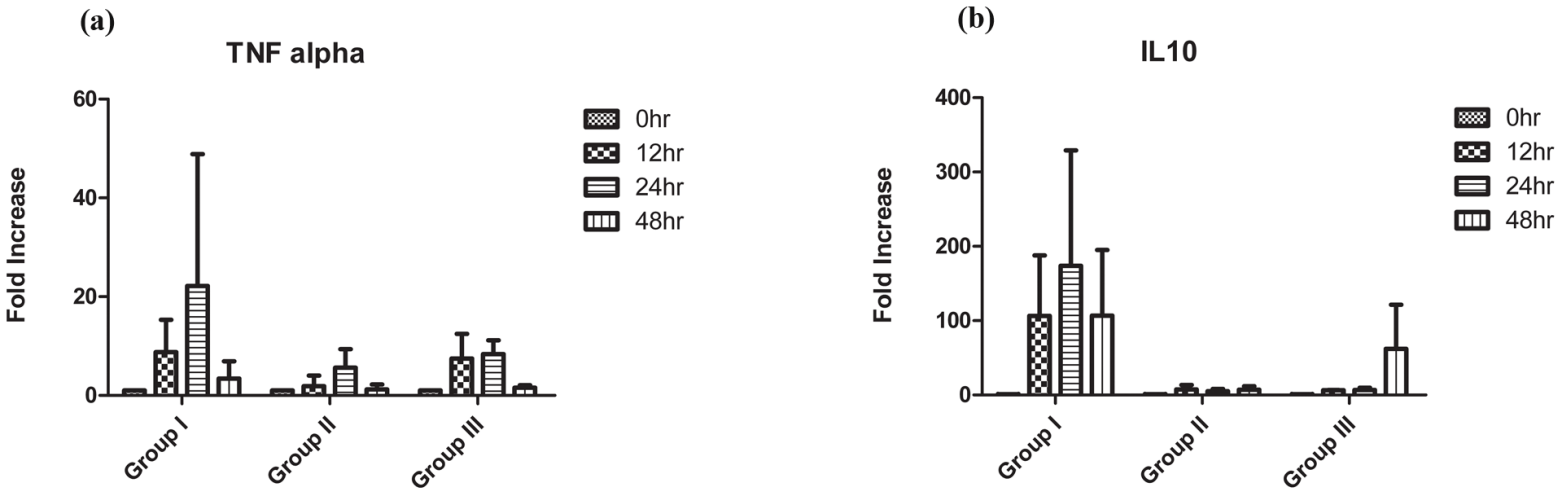
TNFα and IL10 gene expression in koala PBMCs stimulated with UV inactivated *C. pecorum* in Group I, II and III animals. Samples were collected at 0, 12, 24 and 48 hours. Results are expressed as fold increased compared to GAPDH.

PBMCs isolated from all koalas were stimulated with UV-inactivated semi-purified *C. pecorum* elementary bodies for 12, 24 and 48 hours and TNFα and IL0 mRNA expression was measured using our koala specific qrtPCR assays, developed as part of this study. On stimulation with UV inactivated *C. pecorum*, significantly higher mRNA expression levels (*P*<0.05) for IL10 was observed in PBMCs obtained from Group I animals compared to animals in Groups II and III (Figure 4). However, for TNFα mRNA expression a significant difference (*P*<0.05) in expression levels was observed only between Group I and II animals. With one as the baseline for unstimulated PBMCs at 0 hour, a 10 to 50 fold increase was observed in TNFα mRNA expression levels relative to GAPDH at the 48 hours post-stimulation timepoint in Group I animals. Similarly, a 100 to 350 fold increase in IL10 mRNA relative expression (Figure 4) could be observed in the same animals. On average, IL10 mRNA expression levels were observed to be higher (*P*<0.05) than TNFα mRNA expression levels in Group I animals (with current chlamydial disease). In either Group II or Group III animals, no statistically significant difference could be observed between koala TNFα and IL10 mRNA expression, except for the expression (*P*<0.05) of IL10 mRNA at 48 hour time point stimulation which was caused by a high IL10 mRNA expression by an individual animal included in Group III.

## Discussion

Very little is known about the immune system of the koala, confounding efforts to understand and develop strategies to control chlamydial infections in this host. Early studies suggested that the koala is an ‘immunologically lazy’ animal, as delayed humoral immune responses were observed, when compared to placental mammals [34]. However, these reports have not been supported by data from our recent studies evaluating the efficacy of a prototype koala chlamydial vaccine [35], [30] as significant humoral and CMI responses to chlamydial antigens, with similar kinetics, magnitude and duration as seen in other species can be readily detected. A limitation of these studies, however, was an inability to profile the expression of cytokine genes associated with the CMI response since no reagents were available for use in these studies. Hence, the main aim of this study to develop the first reagents to measure cytokine expression in the koala and to provide preliminary data on their response to *C. pecorum* infection.

Before this study, no information was available regrading any koala cytokine gene. Hence, as a first step, nucleotide sequences of the TNFα and IL10 cytokine genes were determined and used to design qrtPCR based assays. The expression of these cytokines was then compared to the housekeeping gene, GAPDH, in order to correct the variation in input RNA in different samples. The efficiency of the qrtPCR assays was shown to be reproducible over subsequent experiments, hence implying the reproducibility of the assays.

The cloned partial sequence of the koala TNFα nucleotide sequence has more than 80% homology to other marsupial TNFα sequences. This level of variability is similar to what is observed among the TNFα sequences of placental mammals (>80%; data not shown). Similar to koala TNFα, the IL10 nucleotide sequence also has more than 80% homology to the two available marsupials IL10 sequences which is comparable to the extent of similarity between the IL10 sequences of the placental mammals. It is this level of conservation at the nucleic acid level of the TNFα and IL10 cytokines among different species, as well as within the marsupials that has enabled their successful cloning in the koala based on the principal of consensus primer design. Hence, this methodology can be employed for the cloning of other koala cytokines for which corresponding marsupial sequences are available. Even though the PCR primers for this assay were designed specifically for koala TNFα and IL10, we cannot exclude the possibility that these may cross-react with other marsupial sequences – a possibility that may open up opportunities for studying related hosts that also lack genomic resources.

In a preliminary study to evaluate the role of these cytokines in koala chlamydial infections and disease, TNFα and IL10 responses of koala PBMCs, isolated from three cohorts of koalas was measured (Figure 4). Although high levels of variability was observed within each group, there was a significantly high expression (*P*<0.05) of TNFα and IL10 mRNA in animals with current chlamydial disease when compared to the remaining animals in the study. Elevated TNFα levels have been observed and associated with conditions such as atherosclerosis and coronary heart disease resulting from *C. pneumoniae* infection [36], [37]. IL10, on the other hand, is an anti-inflammatory cytokine known to play a critical role in chronic infections caused by intra-cellular organisms. During chlamydial infection, high IL10 production has been associated with pathogenesis [38], [39] in a mouse model of *C. trachomatis* infection. Moreover, elevated levels of IL10 have also been observed in women with disease caused by *C. trachomatis* [40], [41]. The observation of high levels of IL10 gene activation in the diseased koalas from Group I may provide the first glimpse at understanding the differences in immune response between asymptomatically infected koalas and koalas with chlamydial disease presentations. In the studies in other hosts, an increase in IL10 production corresponded with a decrease in interferon-gamma (IFNγ) production, which is the principle Th1 cytokine that provides protection against chlamydial infection [42]. IL10 is known to suppress Th1 cytokine response by inhibiting TNFα and IFNγ, the latter two having a synergistic effect on each other [23]. Even though a 300 fold increase in IL10 mRNA expression compared to a 60 fold increase in TNFα expression in koalas with chlamydial disease analysed in this study may be an indication of a similar role played by IL10 expression in koalas as well, a conclusive link can be established only after analysing the IFNγ expression in these animals. No reagents are currently available to measure the latter cytokine, however.

Several factors will need to be considered in future investigations of the koala immune response to chlamydial infection. These include the site of the current chlamydial infection, the genetic diversity of the infected *C. pecorum* strains [26], [30] as well as the age and gender of the animal, beyond the fact that these animals are genetically out-bred. The genetic diversity of *C. pecorum* MOMP, for instance, may partially explain why several animals in Group III produced low levels of TNFα and IL10 expression, respectively, despite being negative by our MOMP-based western blot assays. Other host factors that will also need to be considered include polymorphisms in the IL10 promoter gene and the TNFα, coding sequence which has been shown to affect human immune responses to *C. trachomatis* infection [43], [44]. We did not examine the presence of genetic variation between the IL10 and TNFα sequences between different koalas but this should be a focus for further investigations. The current study analysed *in vitro* TNFα and IL10 production by koala PBMCs on exposure to UV-inactivated *C. pecorum.* However, it is known that chlamydial disease is a manifestation of localised infection and cytokines have a localised mechanism of action. Hence, along with the analysis of more cytokines to better understand the immune response of the koala to chlamydial infection, examining local cytokine production at the site of infection will provide valuable information regarding chlamydial disease in the koala.

## Conclusions

The identification of the first cytokine sequences from the koala and the development of much-needed immunological reagents in this study have provided critical tools and much-needed baseline data on the koala immune system to begin to allow us to elucidate the role of the immune response to infections by this highly pathogenic bacterial infection. Broadening our understanding of the koala immune system is significant due to the ongoing and rapid declines of this native marsupial species across various parts of Australia. Direct and rapid interventions via vaccination of wild koalas is likely required to stabilise declining koala populations from disease threats and these assays, along with the identification of additional koala cytokines, will be critical in evaluating the efficacy of these strategies.

## Acknowledgments

We thank the veterinarians at the Australia Zoo Wildlife Hospital, Beerwah, for helping with koala blood sample collection and Alyce Taylor Brown for laboratory support.
